# Blood-based next-generation sequencing analysis of neuroendocrine neoplasms

**DOI:** 10.18632/oncotarget.27588

**Published:** 2020-05-12

**Authors:** Katerina Zakka, Rebecca Nagy, Leylah Drusbosky, Mehmet Akce, Christina Wu, Olatunji B. Alese, Bassel F. El-Rayes, Pashtoon Murtaza Kasi, Kabir Mody, Jason Starr, Walid L. Shaib

**Affiliations:** ^1^ Department of Hematology and Medical Oncology, Winship Cancer Institute, Emory University, Atlanta, GA, USA; ^2^ Guardant Health, Redwood City, CA, USA; ^3^ Department of Hematology and Medical Oncology, University of Iowa, Iowa City, IA, USA; ^4^ Department of Hematology and Medical Oncology, Mayo Clinic, Jacksonville, FL, USA

**Keywords:** neuroendocrine neoplasm, neuroendocrine tumor, neuroendocrine carcinoma, next-generation sequencing, circulating tumor DNA

## Abstract

**Background:** Neuroendocrine neoplasms (NENs) are a heterogeneous group of neoplasms that span from well-differentiated neuroendocrine tumors (NETs) to highly aggressive neoplasms classified as neuroendocrine carcinomas (NECs). The genomic landscape of NENs has not been well studied. The aim of this study is to confirm the feasibility of next generation sequencing (NGS) testing circulating tumor DNA (ctDNA) in patients with NENs and characterize common alterations in the genomic landscape.

**Results:** Of the 320 NEN patients, 182 (57%) were male with a median age of 63 years (range: 8-93) years. Tumor type included pancreatic NET (*N **=*** 165, 52%), gastrointestinal NEC (*N **=*** 52, 16%), large cell lung NEC (*N **=*** 21, 7%), nasopharyngeal NEC (*N **=*** 16, 5%) and NEC/NET not otherwise specified (*N **=*** 64, 20%). ctDNA NGS testing was performed on 338 plasma samples; 14 patients had testing performed twice and 2 patients had testing performed three times. Genomic alterations were defined in 280 (87.5%) samples with a total of 1,012 alterations identified after excluding variants of uncertain significance (VUSs) and synonymous mutations. Of the 280 samples with alterations, *TP53* associated genes were most commonly altered (*N **=*** 145, 52%), followed by *KRAS* (*N **=*** 61, 22%), *EGFR* (*N **=*** 33, 12%), *PIK3CA* (*N **=*** 30, 11%), *BRAF* (*N **=*** 28, 10%), *MYC* (*N **=*** 28, 10%), *CCNE1* (*N **=*** 28, 10%), *CDK6* (*N **=*** 22, 8%), *RB1* (*N **=*** 19, 7%), *NF1* (*N **=*** 19, 7%), *MET* (*N **=*** 19, 7%), *FGFR1* (*N **=*** 19, 7%), *APC* (*N **=*** 19, 7%), *ERBB2* (*N **=*** 16, 6%) and *PTEN* (*N **=*** 14, 5%).

**Conclusions:** Evaluation of ctDNA was feasible among individuals with NEN. Liquid biopsies are non-invasive methods that can provide personalized options for targeted therapies in NEN patients.

**Patients and Methods:** Molecular alterations in 338 plasma samples from 320 patients with NEN were evaluated using clinical-grade NGS of ctDNA (Guardant360^®^) across multiple institutions. The test detects single nucleotide variants in 54-73 genes, copy number amplifications, fusions, and indels in selected genes.

## INTRODUCTION

NETs are a rare and diverse group of tumors with variable survival outcomes and behaviors [1], defined by tissue-based characteristics that include Ki67 index, grade, and morphology [2]. The annual incidence of NET has been reported to be around 3.65 per 100,000 people, however, due to better diagnostic tools and increased lifespan, the incidence and prevalence of these tumors is on the rise [3, 4]. Although there have been substantial advances in the treatment of NET over the past decade [5], challenges still exist with regard to patient selection and prediction of response. The classification of these tumors is based on tumor grade (assessed by mitotic rate and Ki-67 index) and differentiation: well-differentiated NETs, which are mostly low or intermediate grade, poorly differentiated NECs, which are high grade and aggressive, and the discordant tumors (well differentiated with high tumor grades) [6]. Poorly differentiated NECs often involve multiple sites of metastases, and rarely produce symptoms related to secretion of bioactive substances. Accurate distinction of well-differentiated, indolent tumors from poorly differentiated, aggressive tumors is important since treatment approaches are different with substantial difference in prognosis [7]. The majority of NET are diagnosed at a late stage with around 60–80% presenting with distant metastasis at diagnosis [8]. The 5-year overall survival of patients with NET ranges between 35–82% in well-to moderately differentiated NET and between 4–38% in poorly differentiated NET [3, 9].

With the complexity of the classification, novel biomarkers are required to assist in clinical decision making and ultimately improve patient outcomes [10]. Identification of biomarkers that could be used to guide targets for therapy is an unmet need [11]. Mutational alterations have changed the landscape of treatment in multiple cancers and improved the survival of cancer patients [12–18]. Recently, there has been an increasing interest in circulating tumor DNA (ctDNA) on the basis of studies performed in a range of other cancers [2]. As opposed to traditional tissue biopsies, liquid biopsies are faster, less invasive, have the potential to reflect all metastatic sites (ie tumor heterogeneity), and can indicate therapeutic response or progression through serial sampling [2]. Furthermore, considering the potential of genomic analysis, liquid biopsies offer a facilitated means of detecting genomic alterations and can be easily repeated over time [19–22]. ctDNA testing is now recommended to guide the treatment of lung cancer [23]. A potential challenge that exists with the application of ctDNA in the NEN field is the relative lack of recurrent and/or actionable mutations [2] and this is reported in multiple small studies [2]. Novel technologies such as next generation sequencing (NGS) revealed new molecular aspects of NET over the last years [1, 24]. In a study conducted by Gleeson et al, alterations in MEN1 chromatin remodeling genes and mammalian target of rapamycin (mTOR) pathway genes were found to be the most frequent molecular events identified in pancreatic NET, but it remains unclear whether these biomarkers and other less frequently observed alterations possess predictive value [24]. In this report, analysis of ctDNA through blood-based Guardant360^®^ NGS from patients with a diagnosis of NEN across various histologies is evaluated. The aim was to confirm the feasibility of NGS using ctDNA in NEN and characterize common alterations in the genomic profile. Furthermore, we aimed to identify whether the molecular alterations lead to the identification of potential actionable targets.

## RESULTS

### Patient demographics

**Figure 1:**
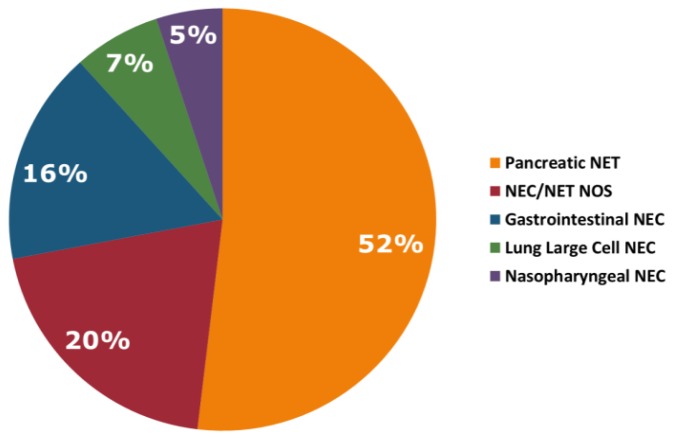
Tumor types.

Between the years 2016 and 2019, a total of 320 NEN patients underwent Guardant360^®^ testing using clinical-grade NGS of ctDNA across multiple institutions, and 280 (87.5%) patients had at least one sample with alterations. The median age was 63 years (range: 8-93), with a male preponderance (57%). Tumor type included pancreatic NET (*N =* 165, 52%), NEC/NET not otherwise specified (NOS) (*N =* 64, 20%), gastrointestinal NEC (*N =* 52, 16%), large cell lung NEC (*N =* 21, 7%) and nasopharyngeal NEC (*N =* 16, 5%) (Figure 1). ctDNA NGS testing was performed on 338 plasma samples; 14 patients had testing performed twice and 2 patients had testing performed three times. A total of 1,012 genomic alterations were identified after excluding variants of uncertain significance (VUSs) and synonymous mutations. Chemotherapy was documented in 48 patients (Supplementary Table 1). The sequence of testing to treatment is unknown. Pathology was obtained in 144 patients and KI-67 score was obtained in 71 patients. KI-67 scores ranged from < 1% to > 99%. Of the 144 patients with documented pathology, 43 patients (30%) were high grade, 8 were intermediate grade (6%), and 3 were low grade.

### Molecular alterations

**Figure 2:**
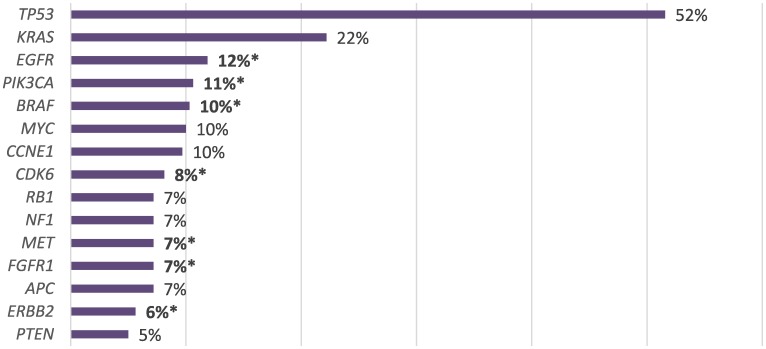
Prevalence of genomic alterations (SM and VUSs excluded) with therapeutic implications (*).

**Figure 3:**
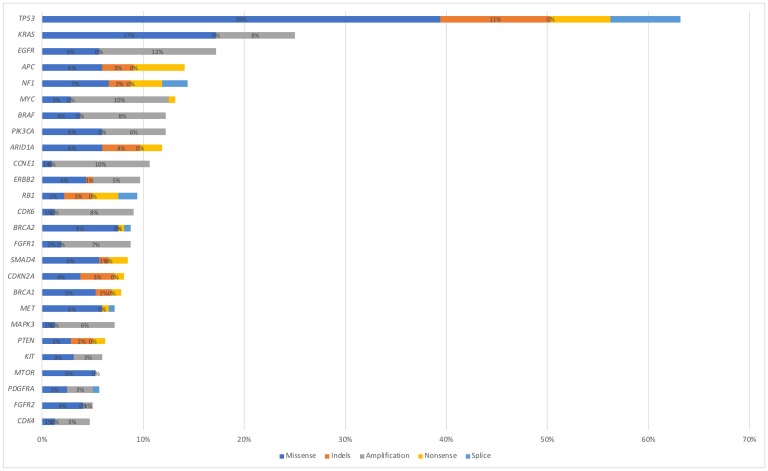
Alteration frequency by gene and alteration type (synonymous alterations excluded).

**Figure 4:**
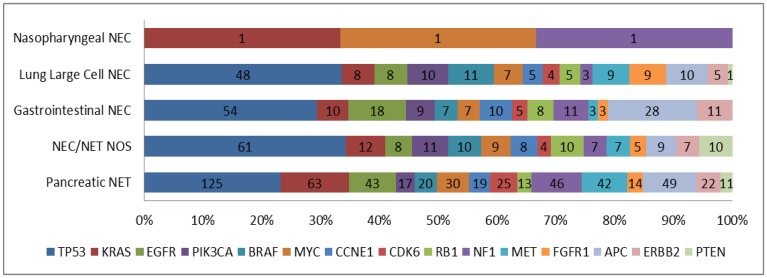
Genomic alterations stratified by tumor type.

In the total cohort of NEN patients, *TP53* associated genes were most commonly altered (*N =* 145, 52%), followed by *KRAS* (*N =* 61, 22%), *EGFR* (*N =* 33, 12%), *PIK3CA* (*N =* 30, 11%), *BRAF* (*N =* 28, 10%), *MYC* (*N =* 28, 10%), *CCNE1* (*N =* 28, 10%), *CDK6* (*N =* 22, 8%), *RB1* (*N =* 19, 7%), *NF1* (*N =* 19, 7%), *MET* (*N =* 19, 7%), *FGFR1* (*N =* 19, 7%), *APC* (*N =* 19, 7%), *ERBB2* (*N =* 16, 6%) and *PTEN* (*N =* 14, 5%) (Figure 2). Alteration frequency by gene and alteration type are shown in Figure 3. Of the 28 patients with BRAF mutations, 3 patients had the V600E alteration. For PNET patients, *TP53* was the most commonly altered mutation (*N =* 125), followed by *KRAS* (*N =* 63), *APC* (*N =* 49), *NF1* (*N =* 46), *EGFR* (*N =* 43), *MET* (*N =* 42), *BRCA1* (*N =* 32), *MYC* (*N =* 30), *BRCA2* (*N =* 29), *CDK6* (*N =* 25), *ERBB2* (*N =* 22), *BRAF* (*N =* 20), *CCNE1* (*N =* 19), *PIK3CA* (*N =* 17), *MTOR* (*N =* 15), *FGFR1* (*N =* 14), *RB1* (*N =* 13) and *PTEN* (*N =* 11). For gastrointestinal NEC patients, *TP53* associated genes were most commonly altered (*N =* 54), followed by *APC* (*N =* 28), *EGFR* (*N =* 18), *ERBB2* (*N =* 11), *NF1* (*N =* 11), *KRAS* (*N =* 10), *CCNE1* (*N =* 10), *BRCA2* (*N =* 10), *BRCA1* (*N =* 9), *PIK3CA* (*N =* 9), *RB1* (*N =* 8), *MYC* (*N =* 7), *BRAF* (*N =* 7), *CDK6* (*N =* 5), *MTOR* (*N =* 4), *MET* (*N =* 3), and *FGFR1* (*N =* 3). For large cell lung NEC patients, *TP53* mutation was most commonly altered (*N =* 48), followed by *BRAF* (*N =* 11), *PIK3CA* (*N =* 10), *APC* (*N =* 10), *MET* (*N =* 9), *FGFR1* (*N =* 9), *EGFR* (*N =* 8), *KRAS* (*N =* 8), *MYC* (*N =* 7), *BRCA2* (*N =* 5), *ERBB2* (*N =* 5), *CCNE1* (*N =* 5), *RB1* (*N =* 5), *CDK6* (*N =* 4), *NF1* (*N =* 3), *MTOR* (*N =* 2), *BRCA1* (*N =* 1) and *PTEN* (*N =* 1). Genomic alterations stratified by tumor type are shown in Figure 4.

### Relationship between age and gender

*KRAS* mutations occurred more commonly among males (66%) with a mean age of 59.3 years. Prevalence of *BRAF* mutations occurred more frequently in males (60%) with a mean age of 61.5 years. Seven *ATM* mutations were detected and occurred more frequently in males (57%) with a mean age of 67.1 years. In this study, *BRCA1* and *BRCA2* mutations were seen in males more frequently (82% and 65%) with a mean age of 54.7 years and 58.9 years, respectively. *MTOR* mutations occurred more commonly in females (56%) with a mean age of 63.4 years and 47 *PIK3CA* mutations were detected, of which 51% were male and mean age was 58.4 years (Table 1). These results need to be validated by a larger sample size in future studies to reach a statistically significant correlation.

**Table 1:** Correlation between age and gender with respect to KRAS/BRAF/ATM/BRCA/MTOR/PIK3CA

Gene	Count of Gene	Male	Female	Mean Age (Years)
KRAS	94	62/94 (66%)	32/94 (34%)	59.3
BRAF	48	29/48 (60%)	19/48 (40%)	61.5
ATM	7	4/7 (57%)	3/7 (43%)	67.1
BRCA 1	45	37/45 (82%)	8/45 (18%)	54.7
BRCA 2	48	31/48 (65%)	17/48 (35%)	58.9
MTOR	27	12/27 (44%)	15/27 (56%)	63.4
PIK3CA	47	24/47 (51%)	23/47 (49%)	58.4

### Plasma-Derived ctDNA for longitudinal disease monitoring

Among the 320 patients studied, 14 patients had testing performed twice and 2 patients had testing performed three times. By analyzing these longitudinal blood samples, we found that new mutations can be gained over time that could potentially be targeted in 11 patients. With serial testing, we identified 1 patient that gained a mutation in *FGFR2*, 1 patient that gained a mutation in *ATM* and 1 patient that gained a mutation in *BRCA1*, which could all be targeted. Loss of mutations was identified in 6 of the 11 patients. These include *TP53*, *KIT*, *RAF1*, *ERBB2, CDK6, PIK3CA, DDR2, CCNE1, NF1, BRCA2, PDGFRA*, and *NOTCH1* (Supplementary Table 3).

## DISCUSSION

NEN represent a heterogeneous group of malignancies varying in biology and behavior. In the era of next generation sequencing the characterization of NEN has led to a better understanding of the molecular underpinnings of these neoplasms [25–28]. As a result, research can be geared towards exploring new pathways to target with both research-based and existing therapies. The current selection of therapies for NETs include somatostatin analogs [29], peptide receptor radionuclide therapy [30], mTOR inhibitors (everolimus), chemotherapy combinations [31] (ie capecitabine/temozolomide) and multi-kinase inhibitors [32] (ie sunitinib, pazopanib, cabozantinib). None of these agents are tailored to select patients on the basis of the presence or absence of molecular alterations, and limited predictive or prognostic biomarkers have been identified other than the location of the primary tumor and the Ki67%. Several translational studies have provided convincing data that epigenetic profiling can identify potential prognostic biomarkers, and some of these have demonstrated preliminary success as serum biomarkers that can be used clinically [33].

With current technology the genome of a tumor can be analyzed by studying the tumor tissue as well as the DNA shed by the tumor (ctDNA). A significant challenge for the use of ctDNA in the NEN field is the relative lack of recurrent mutations in comparison with other tumors. Requirements for accurate ctDNA analysis include adequate tumor DNA being shed into the blood stream and a PCR primer based assay that detects the mutations of interest [34]. Unfortunately, these conditions are only present in a small subset of NET patients population [34]. In small bowel NET, tissue-based genomic sequencing revealed that the majority of recurrent mutations were in cyclin-dependent kinase inhibitor *CDKN1B* (8% of cases) [35]. Pancreatic NET also exhibit recurrent mutations in a relatively limited number of genes, including the tumor suppressor gene *MEN1*, as well as *ATRX* and *DAXX*, genes implicated in chromatin remodeling [36]. Interestingly, mutations in MEN1, DAXX/ATRX or the combination of both MEN1 and DAXX/ATRX were associated with prolonged survival in a study conducted by Jiao *et al*. relative to those patients whose tumors lacked these mutations [36]. The mutational status of DAXX, ATRX and mTOR pathway genes could be used to stratify the prognosis of pancreatic NETs [37]. However, Chan et al. demonstrated contradictory results, whereby mutations in DAXX, ATRX, and MEN1 were associated with adverse clinical outcome in comparison to those without these mutations [38]. This discrepancy between the data could be attributed to a different composition of the tumors. Use of ctDNA analysis in this disease has been inconsistent. Pipinikas et al. established that the ctDNA detected in the blood of 9 tissue samples from 3 pancreatic NET patients had variable concordance with tissue somatic variants [the same tissue somatic variants were detected in ctDNA from cases 1 (*NEBL*) and 3 (*DAXX*)] [39], while Beltran et al. demonstrated that ctDNA and matched tissue biopsies from 64 patients with prostate NET showed approximately 80% concordance [40].

The analysis of ctDNA may be useful for multiple purposes including early detection of residual or recurrent disease, monitoring tumor burden, assessing molecular heterogeneity targeted treatments, prognostic and predictive implications [41]. Recently, Wang et al. demonstrated an *ALK* translocation revealed by ctDNA analysis in a patient with metastatic atypical carcinoid tumor of the lung [42]. The patient was treated with the second-generation ALK inhibitor alectinib with rapid and lasting shrinkage of his disease, supporting the hypothesis that the *ALK* translocation was the driver mutation. In another case report, a patient with high-grade, large cell neuroendocrine cervical carcinoma was successfully treated with nivolumab combined with stereotactic body radiation therapy, based on blood ctDNA results targetting alterations suspicious for high tumor burden [43].

The present analysis is the first and largest population-based study exploring the genetic mutations in patients with NEN utilizing ctDNA derived from liquid biopsy. Some of the alterations reported here are in clinical development as potential targets. In our population of NEN patients, alterations were identified with therapeutic implications that could potentially be targeted by drugs approved for other cancers. Examples of these mutations with their respective frequency of alterations include with possible drug examples: *EGFR* (12%, erlotinib), *PIK3CA* (11%, alpelisib), *BRAF* (10%, vermurafenib), *CDK6* (8%, palbociclib), *MET* (7%, cabozantinib), *FGFR1* (7%, pazopanib, erdafitinib), *ERBB2* (6%, trastuzumab, pertuzumab), and *BRCA1/2* (15%, olaparib). Repeat sampling is a unique advantage of liquid biopsies over tissue based assays. In this series, 16 patients had serial profiling of ctDNA. Analysis showed gain and loss of mutations with time. Some of the gained mutations are targetable including *FGFR2, ATM* and *BRCA1*.

There are several limitations to our study inherent to all retrospective analyses. First, genomics data were obtained from a de-identified database and, hence, only limited clinical information was available. There was no data available regarding whether samples were obtained prior to or after medical treatment/surgery, which limits the interpretation of the analysis. Furthermore, the gene panel is restricted to 73 genes failing to test for *MEN1*, *ATRX* or *DAXX*, which are clinically important in pancreatic NET (Supplementary Table 2). In addition, no survival data was available and the data was limited by the coding of physicians at the different institutions. KI67% and pathology data was lacking in around 50% of patients. It worth noting that our subset of patients did not include specifically small intestinal NETs, which represent the most common NET. It is likely that the majority of the gastrointestinal and the “not otherwise specified” NETs are small intestinal in origin. There is no data to compare tissue genomics to liquid testing in this analysis. Despite these limitations, our findings have important implications. Our study demonstrated that evaluation of ctDNA is feasible among individuals with NEN. Theoretically, as more oncogenic pathways are discovered and more targeted therapies are approved, personalized treatments based on identified unique molecular mutations could lead to improved patient outcomes [44].

Despite the identification of ctDNA as circulating biomarkers capable of providing prognostic information and personalized treatment options in patients with NEN, they have not yet been incorporated into routine clinical practice. More prospective evaluations are required to better understand the role of these biomarkers in NEN, therefore incorporation of ctDNA analysis into clinical trials is highly recommended.

## MATERIALS AND METHODS

This is a retrospective review evaluating the molecular alterations in 338 ctDNA samples from 320 patients who had a diagnosis of NEN and underwent Guardant360^®^ clinical-grade NGS across multiple institutions. The test detects single nucleotide variants in 54–73 genes, copy number amplifications, fusions, and indels in selected genes. Samples from NEN patients between the years 2016 and 2019 were analyzed. Patient-specific covariates included gender and age. Ethical approval was not required for the study; patient identity protection was maintained throughout the study in a de-identified database through a data transfer agreement between Guardant Health and Emory University, and existing data was collected in accordance with the Emory University Institutional Review Board (IRB) guidelines.

### Next generation sequencing

NGS of plasma cfDNA (liquid biopsy) was done by Guardant Health (Guardant360^®^), a College of American Pathologists (CAP)-accredited and Clinical Laboratory Improvement Amendments (CLIA)-certified laboratory. The Guardant360® assay detects single-nucleotide variants (SNV), indels, fusions, and copy number alterations in 73 genes, including the most prevalent tumor suppressor genes in human cancers, with a reportable range of ≥ 0.04%, ≥ 0.02%, ≥ 0.04%, and ≥ 2.12 copies, respectively, as well as microsatellite instability. It does not report tumor mutation burden (TMB). This is a highly analytically/clinically sensitive and specific test, able to detect single molecules of tumor DNA in 10 mL blood samples with an analytic specificity of > 99.9999% [45].

cfDNA was extracted from plasma using the QIAmp Circulating Nucleic Acid Kit (Qiagen, Inc.). Hybrid-capture sequencing libraries were prepared from up to 30ng cfDNA and labeled with nonrandom oligonucleotide barcodes (IDT, Inc.), followed by library preparation, hybrid capture enrichment (Agilent Technologies, Inc.), and sequencing at 15,000 × read depth of the critical exons in the targeted panel by paired-end synthesis (NextSeq 500 and/or HiSeq 2500, Illumina, Inc.). Bioinformatics analysis and variant detection were performed as previously described [46]. NGS data were interpreted by N-of-One, Inc. (Lexington, MA, USA).

## SUPPLEMENTARY MATERIALS





## Abbreviations

NENs: Neuroendocrine neoplasms
NETs: Neuroendocrine tumors
NECs: Neuroendocrine carcinomas
NGS: Next generation sequencing
DNA: Circulating tumor
VUSs: Variants of uncertain significance
mTOR: Mammalian target of rapamycin
IRB: Institutional Review Board
SNV: Single-nucleotide variants
