# Supplementary material for: Blood-based next-generation sequencing analysis of neuroendocrine neoplasms
Source: Oncotarget. 2020 May 12;11(19):1749–57. doi: 10.18632/oncotarget.27588 (PMC7233805; doi:10.18632/oncotarget.27588)
Supplement: Supplementary file 1 [file oncotarget-11-1749-s001.pdf]

Blood-based next-generation sequencing analysis of neuroendocrine neoplasms

SUPPLEMENTARY MATERIALS

Supplemental Table 1: Correlation between mutations and chemotherapy

Chemotherapy	Number of patients treated	Histology	Male	Female	Genes with alterations detected
Cisplatin/Irinotecan	2	PNET, GI NEC	1/2	1/2	CTNNB1, APC, AR, FGFR1, FGFR2, MYC, SMAD4, TP53
Carboplatin/Etoposide	10	PNET, NEC	4/10	6/10	KRAS, TP53, MTOR, AR, PIK3CA, MYC, NF1, CCNE1, ARID1A, CCND2, NF1, CCND1, FGFR1, VHL, RET, SMAD4, BRAF, RB1
FOLFOX	2	PNET	1/2	1/2	NTRK1, PTEN, TP53, ALK
Chemotherapy (CBDCA + PAC…)	1	NEC	0/1	1/1	ARID1A, BRAF, ERBB2, GNAS, MYC, TP53
Afinitor (Everolimus)	5	PNET	4/5	1/5	TERT, JAK2, DDR2, ERBB2, PDGFRA, SMAD4. NOTCH1, VHL, CDKN2A
Octreotide	9	GI NEC, PNET, NEC	5/9	4/9	APC, KRAS, RAF1, TP53, MTOR, GNA11, BRCA1, CDK6, EGFR, KIT, MET, PTEN, ARID1A, ATM, BRAF, ERBB2, FGFR2, HNF1A, MYC, NF1, NTRK3
Lanreotide	4	PNET, NEC, GI NEC	0/4	4/4	TP53, JAK2, GNAS, CCND2, CDKN2A, ERBB2, KRAS, MTOR
AZD 1775/Olaparib	1	NEC	0/1	1/1	TP53, MET, ALK
Carboplatin	1	NEC	0/1	1/1	CTNNB1, TERT
Cisplatin/Etoposide	5	NEC, PNET, Lung large cell NEC	2/5	3/5	TP53, APC, BRCA2, CCNE1, KRAS, NF1, FGFR2, MTOR, PDGFRA, EGFR, ERBB2, FGFR1, KIT
Taxol/Carboplatin	3	NEC, GI NEC, Lung large cell NEC	2/3	1/3	ARID1A, BRCA2, CCNE1, EGFR, ERBB2, PIK3CA, TP53, APC, BRAF, BRCA1, CCND2, FGFR1, KRAS, MYC, PDGFRA, FGFR3, GNAS, NF1, RB1, TERT
S/P platinol + etoposide, S/P Keytruda, S/P Topotecan	1	NEC	1/1	0/1	CDKN2A, RB1, TP53, EGFR, ARID1A, BRCA1, CDKN2A, FGFR2, NF1, NOTCH1
Gemzar/Abraxane	3	PNET	2/3	1/3	AR, KRAS, NF1, TP53, APC, GNAS, SMAD4

Capecitabine + Temozolomide	1	PNET	1/1	0/1	VHL
FOLFIRINOX	2	PNET	1/2	1/2	NF1, TP53, ATM, KRAS, SMAD4
Onivyde/5FU	1	PNET	0/1	1/1	FGFR1, KRAS

Supplementary Table 2: Genes sequenced by Guardant360 and all four major classes of alterations

Point Mutations (SNVs) (73 Genes)	Indels (23 Genes)	Amplifications (18 Genes)	Fusions (6 Genes)
AKT1 SMO ARAF	*ATM*	*AR*	*ALK*
ATM APC CCND1	*ARID1A*	*CCND1*	*FGFR2*
CCNE1 BRCA1 CDKN2A	*BRCA2*	*CCNE1*	*FGFR3*
DDR2 CDK4 EZH2	*CDKN2A*	*CDK6*	*NTRK1*
FGFR1 ERBB2 (HER2) GNA11	*ERBB2*	*ERBB2*	*RET*
GNAS FGFR3 IDH2	*KIT*	*FGFR2*	*ROS1*
JAK3 HRAS MAP2K2/MEK2	*MLH1*	*KRAS*	
MAPK3/ERK1 KRAS MTOR	*NF1*	*MYC*	
NF1 MLH1 NRAS	*PTEN*	*PIK3CA*	
RB1 NOTCH1 PTPN11	*SMAD4*	*BRAF*	
SMAD4 PIK3CA RIT1	*TP53*	*CCND2*	
VHL RHEB TP53	*VHL*	*CDK4*	
ALK STK11 ARID1A	*APC*	*EGFR*	
BRAF AR CCND2	*BRCA1*	*FGFR1*	
CDH1 BRCA2 CTNNB1	*CDH1*	*KIT*	
EGFR CDK6 FBXW7	*EGFR*	*MET*	
FGFR2 ESR1 GNAQ	*GATA3*	*PDGFRA*	
HNF1A GATA3 JAK2	*MET*	*RAF1*	
KIT IDH1 MAPK1/ERK2	*MTOR*		
MET MAP2K1/MEK1 MYC	*PDGFRA*		
NFE2L2 MPL NTRK1	*RB1*		
PDGFRA NPM1 RAF1	*STK11*		
RET RHOA ROS1	*TSC1*		
PTEN TERT SC1			
NTRK3			

Supplementary Table 3: Longitudinal study of genomic alterations in repeated blood samples. See Supplementary Table 3
