# Supplementary material for: Blood-based next-generation sequencing analysis of neuroendocrine neoplasms
Source: Oncotarget. 2020 May 12;11(19):1749–57. doi: 10.18632/oncotarget.27588 (PMC7233805; doi:10.18632/oncotarget.27588)
Supplement: Supplementary file 2 [file oncotarget-11-1749-s002.docx]

Supplemental Table 3: Longitudinal study of genomic alterations in repeated blood samples

| **Patient Number**  **(n=11)** | **Date of blood draw** | **Gene** | **Alteration** | **Percentage (%)*** |
| --- | --- | --- | --- | --- |
| **1** | November 2016  ------------------------  December 2016 | TP53  TP53  PIK3CA  PIK3CA  PIK3CA  KIT  ESR1  APC  APC  ------------------------  PIK3CA  PIK3CA  NF1  ESR1  APC  APC | K291N  G293G  E545K  E542K  A1066V  D975N  L525L  E2655K  E243*  ------------------------  E545K  A1066V  S2384C  L525L  E2655K  E243* | 0.15337056  0.14314993  16.442616  0.47032855  0.3223231  0.13489727  2.7007372  0.50332872  15.576422  ----------------------  26.827538  1.0753636  0.16265253  8.9091128  0.2897671  25.895538 |
| **2** | January 2017  ------------------------  March 2017 | KRAS  GNAS  APC  ------------------------  SMAD4  KRAS  GNAS  APC | G12V  R201H  p.Lys1310fs  ------------------------  H132P  G12V  R201H  p.Lys1310fs | 41.765864  47.56266  49.470631  ----------------------  33.090951  37.895011  44.446414  40.327694 |
| **3** | September 2017  ------------------------  March 2018 | TP53  RAF1  KRAS  ERBB2  CDK6  CCNE1  ------------------------  TP53  KRAS  CCNE1 | V272M  AMP  G12D  AMP  R87R  AMP  ------------------------  V272M  G12D  AMP | c.814G>A  PCN 2.28  c.35G>A  PCN 2.29  c.259C>A  PCN 2.99  ----------------------  13.8251  8.80904  PCN 2.23 |
| **4** | March 2017  ------------------------  July 2017 | KRAS  CTNNB1  CDK6  BRAF  ------------------------  NF1  KRAS  FGFR2  EGFR  CTNNB1  CDK6  BRAF  ARID1A | AMP  S45F  AMP  G466R  ------------------------  p.Asp372fs  AMP  I563L  AMP  S45F  AMP  G466R  p.Ser1948fs | PCN 2.41  21.833607  PCN 2.46  0.38353927  ----------------------  0.0934394  PCN 2.73  0.117352  PCN 2.85  45.7074  PCN 3.17  0.0967075  0.0316355 |
| **5** | May 2017  ------------------------  September 2017 | TP53  SMAD4  NF1  ------------------------  TP53  SMAD4  NF1 | p.Pro153fs  V465M  F1193C  ------------------------  p.Pro153fs  V465M  F1193C | 0.061447296  0.13706578  0.18631371  ----------------------  0.06192  0.156704  0.27284 |
| **6** | February 2017  ------------------------  October 2017 | TP53  PIK3CA  ERBB2  DDR2  CCNE1  APC  ------------------------  GNA11  ATM  APC | Y126H  AMP  AMP  R668H  AMP  p.GIn1123fs  ------------------------  N222K  R337C  p.GIn1123fs | 24.611235  PCN 2.39  PCN 2.37  0.35267122  PCN 2.43  18.382211  ----------------------  0.540992  0.359187  0.136553 |
| **7** | June 2017  ------------------------  December 2017 (early)  ------------------------  December 2017 (late) | NF1  MTOR  BRCA2  ------------------------  PDGFRA  MTOR  KIT  ESR1  CDK6  ------------------------  TSC1  MTOR  ESR1  BRCA2 | R135Q  G1488E  N2427N  ------------------------  AMP  G1488E  P341S  S566N  AMP  ------------------------  P1145S  G1488E  S566N  N2427N | 0.128995  0.415824  0.0955369  ----------------------  PCN 2.21  0.191247  0.189799  0.16266  PCN 2.19  ----------------------  0.447766  1.03841  0.150964  0.546572 |
| **8** | June 2017  ------------------------  December 2017  ------------------------  September 2018 | VHL  ------------------------  VHL  ------------------------  VHL | R167W  ------------------------  R167W  ------------------------  R167W | 10.172  ----------------------  11.1531  ----------------------  9.14 |
| **9** | March 2017  ------------------------  August 2017 | TP53  TP53  EGFR  CCNE1  BRCA2  ------------------------  TP53  TP53  EGFR  CDK6  CCNE1 | Y205C  p.Ala347fs  AMP  AMP  V2050I  ------------------------  Y205C  p.Ala347fs  AMP  AMP  AMP | 46.267101  32.183388  PCN 3.24  PCN 3.57  27.865365  ----------------------  38.2428  26.2287  PCN 2.80  PCN 2.82  PCN 2.79 |
| **10** | October 2018  ------------------------  November 2018 | TP53  TERT  APC  ------------------------  TP53  TERT  HNF1A  APC | R196P  NA  S1327*  ------------------------  R196P  NA  L307M  S1327* | 47.96  4.38  29.94  ----------------------  27.46  3.01  13.14  16.13 |
| **11** | December 2017  ------------------------  July 2018 | PDGFRA  NOTCH1  ------------------------  CDK6  BRCA1 | AMP  A1552A  ------------------------  AMP  Y112F7 | PCN 2.27  0.164908  ----------------------  PCN 2.21  0.118389 |

*Percentage refers to the number of genomic alterations/variants found in a particular gene out of the total number of genomic alterations detected in 320 patients.

PCN = Plasma Copy Number
